# Renal Gene Expression Database (RGED): a relational database of gene expression profiles in kidney disease

**DOI:** 10.1093/database/bau092

**Published:** 2014-09-24

**Authors:** Qingzhou Zhang, Bo Yang, Xujiao Chen, Jing Xu, Changlin Mei, Zhiguo Mao

**Affiliations:** Kidney Institute of CPLA, Division of Nephrology, Changzheng Hospital, Second Military Medical University, 415 Fengyang Road, Shanghai 200003, China

## Abstract

We present a bioinformatics database named Renal Gene Expression Database (RGED), which contains comprehensive gene expression data sets from renal disease research. The web-based interface of RGED allows users to query the gene expression profiles in various kidney-related samples, including renal cell lines, human kidney tissues and murine model kidneys. Researchers can explore certain gene profiles, the relationships between genes of interests and identify biomarkers or even drug targets in kidney diseases. The aim of this work is to provide a user-friendly utility for the renal disease research community to query expression profiles of genes of their own interest without the requirement of advanced computational skills.

**Availability and implementation:** Website is implemented in PHP, R, MySQL and Nginx and freely available from http://rged.wall-eva.net.

**Database URL:** http://rged.wall-eva.net

## Introduction

Kidney diseases are among the major public health burdens globally and associated with increased risk of cardiovascular morbidity and mortality, resulting in poor prognosis (1, 2). The pathogenesis of most kidney diseases are associated with the interplay of genetic predisposition and environmental factors; however, discovering the functional links to bridge the gap from genetic risk loci to disease phenotype is challenging (3). Many strategies like genome-wide association study, microarray expression profiling and gene sequencing were used to identify kidney disease-associated genetic risk factors and better understand the molecular mechanisms underlying the pathogenesis of kidney diseases, hence produced large amounts of bioinformatics data (4).

DNA microarray technology aims at simultaneous measurements of the expression of thousands of genes in one single experiment. Over the past few years, this technology allowed better understanding of the complex and heterogeneous molecular characteristics of renal tissues and helped to make individualized therapeutic strategies in kidney diseases (5). Recently, high-throughput sequencing of cDNA (RNA-seq) has provided a powerful alternative for mapping and quantifying transcriptomes and showed its advantages in many aspects (6, 7). However, for most published DNA microarray and RNA-seq studies, only a subset of genes of the researchers’ interests were reported to demonstrate their academic hypothesis. The complete data sets uploaded are stored in an unsystematic manner, and seem merely useful to those with computational expertise, whereas for most researchers it remains difficult to handle when answering scientific questions.

Here we present Renal Gene Expression Database (RGED), a database of gene expression profiles generated from high-throughput DNA expression profiling experiments in kidney diseases research. The database is provided with an integrated web-based utility, which made the data easily accessible to the kidney disease research community. User can investigate the expression profile of a certain gene in kidney diseases and analyse the correlations between two genes of interests (positively or negatively related, how close they are related). As the closely correlated genes are suggested to share a similar regulatory pathway, this feature of the database might provide potential research proposals on the mechanisms of kidney diseases.

## Database contents

We searched NCBI Gene Expression Omnibus (GEO) database (8) for gene expression profiling experiments with the keyword of kidney diseases. A total of 88 studies, published between 2004 and 2014, were selected for further processing. As shown in Table 1, the studies included in this database focused on several major kidney diseases, including acute kidney injury, autosomal dominant polycystic kidney disease, chronic kidney disease, IgA nephropathy and kidney carcinoma. Because these experiments were conducted by different research teams, the assay platforms varied. Three major providers of the experiment platforms are Affymetrix, Inc., Illumina, Inc. and Agilent Technologies, Inc.

**Table 1.** List of DNA microarray experiments by kidney disease state

Kidney disease state	Number of experiments
Acute kidney injury	2
Autosomal dominant polycystic kidney disease	1
Chronic kidney disease and the uremia	5
Diabetic nephropathy	8
IgA nephropathy	3
Kidney carcinoma	39
Kidney transplantation	28
Lupus nephritis	1
Nephrosclerosis	1

**Figure 1.**
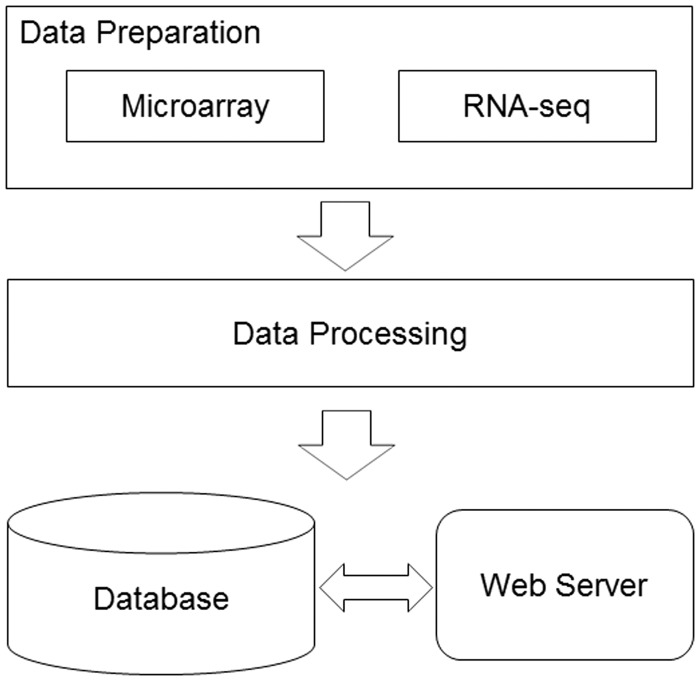
The database construction pipeline. The diagram demonstrates the work flow in the RGED development. In the data preparation step, data sets generated by two technologies, DNA microarray and RNA-seq, were considered; in the data processing step, two bioinformatics pipelines were used to normalize and annotate the gene expression data; all the data were deployed in the MySQL database, while the web server provided the user interface.

**Figure 2.**
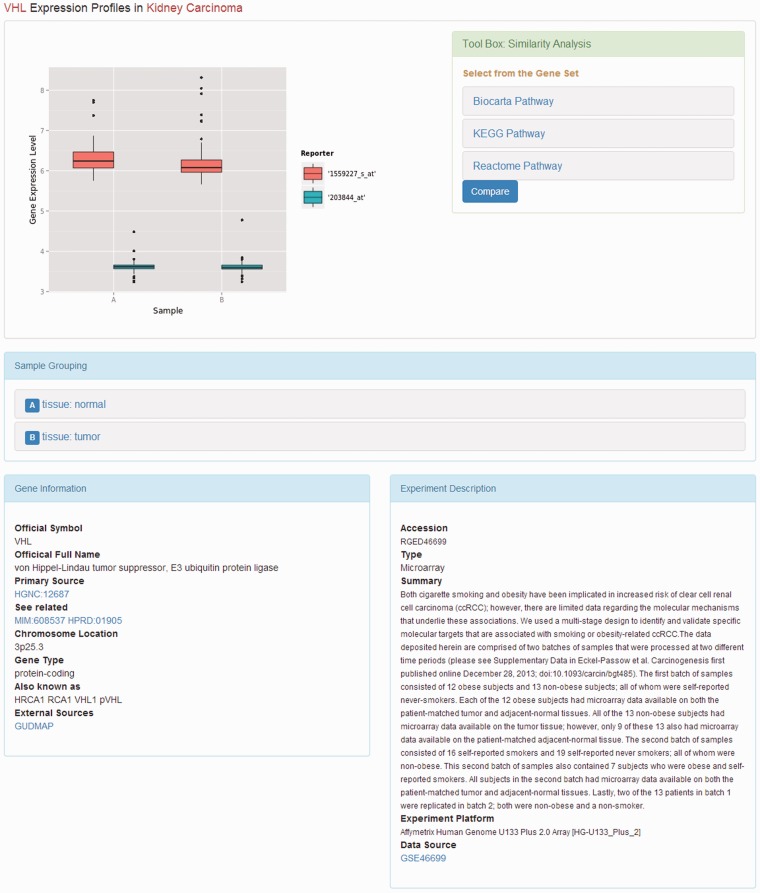
Web page of gene expression profile. The web page displays the gene expression profiles. The description of the experiment and the subgrouping of the samples are placed under the box plot of the gene expression levels. The tool box providing similarity analysis is on the right side of the box plot.

We developed a bioinformatics pipeline (Figure 1) for the data preprocessing before implanting the web server. For DNA microarray experiments, first, the expression data sets, the series matrix files, were downloaded from the GEO ftp site. Second, the R/Bioconductor package, GEOquery (9), was used to load the series matrix files and process the 2-based log transformation to make the data comparable. During this process, the reporters with null or zero expression values will be dismissed. Third, the annotation files were also retrieved from GEO database using the GPL accession number. Finally, each reporter on the microarray was mapped to the available official gene symbol to make the database easier for query. For RNA-seq experiments, the raw reads data were retrieved from the European Nucleotide Archive (10). The RNA-seq data analysis pipeline was introduced to process the data sets before they were deployed in the database. The software used in the pipeline included Bowtie2 (11), Tophat (12) and Cufflinks (13). The database can be freely accessed at http://rged.wall-eva.net. The web application is written in PHP and Javascript. The database runs a MySQL engine (innoDB) on an Ngnix Server (http://nginx.org/). When requested by the client web browser, the R package, ggplot2 (14), was implemented to visualize the gene expression levels (Figure 2).

For particular experiments that contain only two groups of samples, such as normal state versus disease state and tumour adjacent tissue versus primary tumour tissue, we conducted differentially expressed gene analysis. By doing this, the potential genes of interest would be highlighted to the users for further analysis. The R/Biocondutor package, limma (15), was implemented. During this process, the empirical Bayesian methods are used to provide stable results. The linear model and differential expression functions were applied to all microarray data sets. The genes were identified as significantly expressed based on the threshold of *P-value* <0.01 and |logFC|>1. To eliminate misleading, we selected top 100 of these genes and listed them under the summary section of each corresponding experiment.

One important feature of this database is that it enables users to look for gene candidates with closely related to expression pattern of the genes interested, suggesting the existence of common regulatory pathway between these genes. By which, researchers may find possible novel gene functions in molecular signals regulations. The gene sets predefined in the database were retrieved from various sources. The gene set consists of candidates from KEGG (16), BIOCARTA (www.biocarta.com) and REACTOME (17).

When the gene expression similarity search was requested, a Perl script was used to generate the matrix containing gene expression data from the local MySQL database. In the expression matrix, each gene corresponds to one row and each sample corresponds to one column. The hierarchical clustering algorithm was introduced to determine the relationships among genes. This attempt was processed using a combination of distance metrics and linkages. The R package, pheatmap, was called to implement the clustering algorithm and then plot the results (Figure 2).

Currently, the RGED release 1.0 has a collection of 5354 samples, including cell lines, model animals and human tissues. Among the 88 studies integrated in this database, 39 experiments are about the kidney carcinoma researches and 28 experiments focus on kidney transplantation (For more details, look into the data sets).

## Website

The main purpose of RGED is to help researchers to search gene expression profiles across various kidney disease states. The design of the web interface for the database enabled users search across the database easily without need of computer expertise. The home page of the website provides two ways to query the database: one is to search the gene of interest by inputting keyword, the other one is to browse the data sets by disease classification. Users can also simply click on the tags of some popular genes to start a quick search.

When the user clicks the link of a kidney disease, the available information of the study on that disease will be displayed. On this page, the summary and published year of the DNA microarray experiments are presented along with the list of top regulated genes if available. The listed genes are sorted by *P*-value. By clicking on the gene symbol, more details about the gene expression profiles in the kidney diseases will be returned (Figure 2).

**Figure 3.**
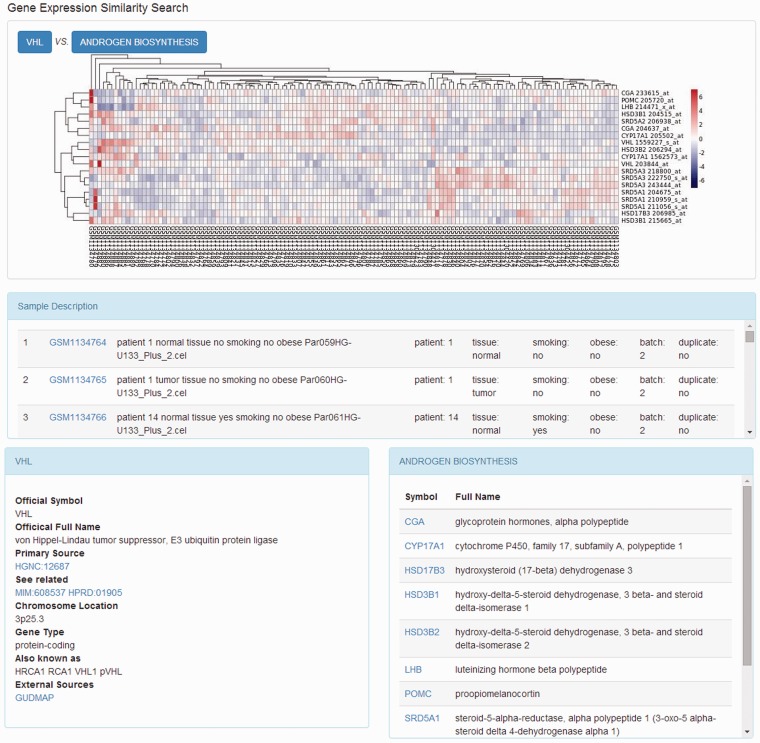
Heat map of genes expression relationship. The heat map shows the clustering results of the gene expression pattern comparison.

On the page that displays the expression profile of a gene, there is a box plot indicating the expression levels of the gene in the samples from different subgroups. The descriptions of the experiment as well as the samples are presented along with the plotted graphics. The information including official symbol, official full name, primary source, chromosome location, gene type and synonymous names are provided in the Gene Information box. To enable users further investigate the gene, there are also links to other sources, including OMIM (18), the Ensembl (19) database, HPRD (20), the Vega database (21) and GUDMAP (22). Moreover, on the top right of the page, there is a toolbox where the user can compare the expression patterns against other genes from certain pathways, such as AKT pathway, or apoptosis. The comparison result is displayed as a heat map, where the genes with the most similar expression patterns are placed next to each other, suggesting the close relationship between these genes (Figure 3). In this page, the list of genes analysed was provided with links to more information.

## Conclusion

In this study, we present a relational database of gene expression profiles in kidney diseases so as to establish a user-friendly interface to query genome-wide expression profiles in various renal cell lines and human kidney tissues for the research community.

To date, a few databases containing the kidney genes expression have been set up. Polycystic Kidney Disease Mutation Database collects autosomal dominant polycystic kidney disease gene variant data exclusively (23). Another database, the Kidney Development Database provides information on gene expression during the development of the pro-, meso- and metanephroi of a range of vertebrates (24). Although Kidney Gene Database (http://www.urogene.org/kgdb) is a potentially useful database of genes including data from various kidney diseases (25), the constructers stopped updating it since 2004. In addition, this database only contains genes or genomic loci involved in human, which may not address the needs of kidney disease research sufficiently. Our study made a promising leap forward in collecting, managing and publishing the gene expression data sets in the area of kidney research. Using RGED properly, researchers could explore certain gene profiles, the relationships between genes of interests and identify biomarkers or even drug targets in kidney diseases.

In the future, we will continue collecting gene expression data sets from published renal disease research using DNA microarray and RNA-seq technologies, to keep RGED updated. We will also develop new online utilities to help users query, investigate and analyse genes of their interests in kidney diseases.

## Funding

This work was supported by Chinese Society of Nephrology (No.13030340419) and the national Key Technology R&D Program (No.2011BAI10B08). Funding for open access charge: 800 GBP.

*Conflict of interest*. None declared.
